# Moral judgment of genetic technologies: validation of the genetic technologies questionnaire in the German-speaking population

**DOI:** 10.3389/fgene.2025.1620962

**Published:** 2025-08-01

**Authors:** Birgit Teichmann, Florian Melchior, Konrad Beyreuther, Maria K. Chorianopoulou

**Affiliations:** ^1^Network Aging Research (NAR), Heidelberg University, Heidelberg, Germany; ^2^Department of Philosophy, National and Kapodistrian University of Athens, Athens, Greece

**Keywords:** genetic technologies, genome editing, genetic testing, ethics, knowledge, moral status, autonomy, religiosity

## Abstract

**Introduction:**

The development of modern life sciences has expanded our biomedical capabilities to an unprecedented degree. For example, genetic testing can be used to predict hereditary predisposition or susceptibility to certain diseases. The development of gene scissors such as CRISPR/Cas makes it possible to repair the disease gene or introduce a protective gene in somatic cells but also in germline cells, leading to permanent changes of the genome. But is everything we “can” do morally justifiable? To what extent does the moral status of the living being, autonomy, and privacy influence the decision of whether something is morally “good” or “bad”? There is a lack of valid instruments to study the moral judgment of genetic technologies. Therefore, the aim of this study is to translate and validate the “Genetic Technologies Questionnaire” (GTQ) and the short version of the “Conventional Technologies Questionnaire” (CTQ5) into German.

**Methods:**

Convenience sampling (N = 317) was used to conduct a cross-sectional online study. Analyses included internal consistency, structural validity, known group construct validity, tests for floor and ceiling effects, and retest reliability with a subset of n = 69. Correlational analyses were conducted with education, age, prior knowledge of genetics, religiosity, conventional technologies, and prior genetic testing. This study used the STROBE checklist for reporting.

**Results:**

The GTQ30 (Cronbach’s α = 0.938) and GTQ20 (α = 0.940) are reliable and stable instruments for testing the moral judgment of lay people, while the GTQ5 (α = 0.857) and CTQ5 (α = 0.697) showed some weaknesses. Conventional technologies were judged morally better than genetic technologies, and genetic testing considered better than genome editing. Two additional versions were validated: the GTQ-Human (GTQ-H), using all items relating to humans, and the GTQ-Moral Status (GTQ-MS), including one item per different group of living beings for genetic testing and one for genome editing.

**Conclusion:**

The GTQ is a valid instrument that is now available in shorter versions for different areas of research: the GTQ-MS for philosophical questions addressing moral status and the GTQ-H for biomedical and psychological questions related to research, prognosis, diagnosis, and therapy in humans.

## Introduction

Since the discovery of DNA by Watson and Crick in 1953 (WATSON and CRICK, 1953) – but at least since the Human Genome Project (1990–2003), which has made it possible to decode more than 90% of human DNA (Collins et al., 2003) – genetic technologies have developed rapidly. This is opening up new applications in areas such as research, medicine, and agriculture that continue to challenge our moral intuitions and provoke ethical and legal debates.

Genetic tests are biochemical and molecular biological methods that can be used to detect changes in gene sequence (deletions, additions, or misspellings) or in the level of expression. They may also refer to the microscopic analysis of chromosomes (AMA, 2022). Genotyping can provide valuable indicators of disease diagnosis, prognosis, and progression and guide selection and response to therapy (Ishida et al., 2024). Other applications include forensics, parentage studies, and the study of inheritance within populations (Kling et al., 2021).

However, while genetic testing has the potential to improve healthcare, it also raises ethical issues, primarily related to patient and physician education and counseling, privacy and confidentiality, and distributive justice. In addition, genetic data are “family data,” which means that the data can potentially provide information about the disease risk of family members. This poses a dilemma for both the clinician and the patient as to whether the information should be shared or even whether there is a duty to do so–at least in cases where timely treatment can halt the progression of the disease–a decision that should also take into account the “right not to know.” In particular, predictive tests for diseases for which there is currently no prevention or treatment pose a major ethical challenge to the clinician (Lehmann et al., 2022).

The societal implications of predictive genetic testing, genetic screening, and increasing knowledge about the function and interaction of genetic factors are manifold. For example, they raise expectations among patients. Evidence of the effectiveness of prevention is problematic; however, the activities of informed individuals to reduce the risk of disease also seem to have limitations, while more responsibility for health is placed on the patient. In addition, genetic testing carries the risk of genetic discrimination, which could affect both employment and insurance, depending on national laws and health systems (Kollek and Lemke, 2008).

The advent of targeted gene editing technologies, especially the development of gene scissors such as CRISP/Cas9 (Jinek et al., 2012), ushered in a new era of precision medicine. It became possible not only to detect errors in DNA sequences but also to correct specific disease-causing mutations in already differentiated somatic cells as well as in germline cells, i.e., both germ cells and single-cell embryos, thereby limiting genetic diseases in offspring as well as in future generations (Ryu et al., 2023). In the few years since its discovery, more than 110 gene-editing-based therapies are now in clinical trials, with treatments for sickle cell anemia and Thalassemia already in phase 3 (CRISPR Medicine, 2023). No therapy based on genome editing is currently approved in Germany. In addition, the German Ethics Council has decided that the techniques of germline therapy through off-target and on-target mutations are still too uncertain and, therefore, ethically irresponsible (Deutscher Ethikrat, 2019).

In addition to its use in human medicine, genome editing has rapidly become established worldwide in the research and development of new plant varieties (Wada et al., 2020) and in farm animal breeding. While previous research on genome editing in farm animals has focused on disease resistance (e.g., tuberculosis), production, allergen elimination, and animal welfare traits, the use of genome editing to introduce beneficial alleles (e.g., heat tolerance, disease resistance) has the potential to maintain or even accelerate genetic gains achieved through conventional breeding programs, thereby increasing productivity (van Eenennaam, 2019). Similarly, CRISPR technology has already been successfully used in plant breeding to improve stress tolerance, selfing, nutritional value, flavor, and metabolic products (Sharma et al., 2023). Whether and to what extent the products can be commercialized depends on national regulations, with genetic engineering legislation imposing very high hurdles and strict proof requirements for the marketing and release of genetically modified organisms (Sprink et al., 2022).

While agricultural genome editing is mainly based on utilitarian principles, i.e., the assessment of consequences and “the greatest good for the greatest number” (Bentham, 2013), Tom I. Beauchamp and James F. Childress developed the four basic principles of ethical practice that have become established in medical ethics: respect for autonomy, the principle of non-maleficence, beneficence and justice (Beauchamp and Childress, 2019). Issues of autonomy arise primarily in relation to interventions on the embryo, which cannot make decisions for itself. The principles of non-maleficence and beneficence concern not only the patient *per se* but should always be considered in relation to future generations, while the principle of justice applies to all of the above. Equal access to the relevant genetic technologies is therefore of great importance, as is fair treatment of individuals regardless of their genetic profile (Joly et al., 2014). Even if germline interventions were considered ethically legitimate in principle, the associated risks would initially only be accepted for the prevention of serious diseases without treatment alternatives (Deutscher Ethikrat, 2019).

To date, there is a paucity of research that examines the ethical judgment of the general population on genetic technologies. The following questions are important if training is to be provided to motivate the general population to participate in political debates: Is basic genetic knowledge necessary; what do citizens know about gene-environment relationships or is the belief in genetic determinism still widespread; does the moral status of plants, animals, and humans contribute to different moral judgments; is there a difference in moral status between embryos and adults?

The moral status of living beings refers to the question which living beings are protected by moral norms and why, i.e., what is the reason for moral status (Shea, 2018). In general, the concept of moral status tells something about how morally significant a creature is, whether one is morally bound to consider its needs, interests, or welfare (Warren, 2004). The higher the moral status, the more its interests count in moral evaluation (Jaworska and Tannenbaum, 2013). This notion has implications for moral agents, i.e., whether and to what extent they have a moral obligation to the living being and whether they are morally obliged to consider its needs, interests, or welfare. Most ethicists–in particular the preference utilitarian Singer – would ascribe a higher moral status to certain species of animals than to a human embryo or a person with dementia because they have more complex cognitive capacities (Singer, 2011). An embryo may have a different moral status from an adult since it has the potential to develop and belongs to the species *homo sapiens* but cannot make decisions about its own future. Therefore, an embryo is the subject of moral rights but not duties (Shea, 2018).

There are two main approaches to assigning moral status: the utilitarian approach, in which one’s own interests, such as pain and pleasure, are included in the calculation of “the greatest happiness for the greatest number” (Bentham, 2013), and the non-utilitarian approach, which takes into account reasons to act in the interest of the entity (Jaworska and Tannenbaum, 2013). Status issues are of utmost relevance when it comes to issues such as embryo research, the use of animals as food, for biomedical research, or even as producers of human organs, thus using animals “only as a means and not as an end,” which also contradicts Kant’s categorical imperative (Shea, 2018; Kant, 2019).

The moral assessment of biotechnologies and their applications has been changing over time. Just as the birth of the first “test-tube” baby was heavily criticized in 1978, *in vitro* fertilization is now routine worldwide (Fauser, 2019). This radical change makes it even more important to provide information that enables the general public to participate in social consensus-building and, ultimately, to provide a (moral) basis for the establishment of normative rules (laws, conventions, decision-making bases for ethics committees) for the responsible treatment of life.

Although there is a growing number of studies on public opinion regarding gene therapy and genome editing (Delhove et al., 2020) and the factors that influence public opinion (McCaughey et al., 2019), there are hardly any empirical studies on the moral judgment of genetic technologies. Unlike opinions or attitudes, moral values are not easily changed, as they are based on values and norms that are accepted by society as a whole or by certain subgroups of society (Agostini, 2021) and change only slowly over time in response to social and cultural transformation (Kolster, 2013). The aim of the present study is, therefore, to translate and validate the “Genetic Technologies Questionnaire” by Küchenhoff et al. (2022) into German. The questionnaire was developed to assess the moral judgment of laypersons on genetic testing and genome editing and contains six categories: genetic testing of humans and non-humans, genome editing of humans and non-humans, data protection, and social justice. The questions were chosen to address living beings of different moral status–embryos, adults, animals, and plants–and to vary in the severity of the intervention–testing *versus* editing. The original questionnaire was designed to be used in its full 30-question format, but it is also available in a 20-question and a five-question version, which still includes the four categories of living beings, but only questions about genome editing and none about genetic testing.

The questionnaire was developed using a contrasting design, i.e., for each item, there is an almost identical item by replacing genetic technology with conventional technology (Conventional Technologies Questionnaire (CTQ) e.g., “genetic test” with “ultrasound scan.” In the present study, only the five items contrasting with the GTQ5 version were included.

The present study aims to examine the psychometric properties of the translated questionnaires and to test the following hypotheses: Conventional techniques will be judged morally better than genetic technologies. Genetic testing will be judged morally better than genome editing. Human genome editing is morally worse than non-human genome editing. Genome editing of embryos is morally worse than that of adults. Religious people judge genetic technologies to be morally worse than non-religious people. Moral judgment depends on knowledge of genetics, which in turn depends on age and level of education. In addition, variants of the questionnaire should be tested. One variant should include questions about humans only to enable its use in human genetics. Another variant should include questions about gene testing and gene editing in different living beings to investigate differences in moral status.

## Materials and methods

The final sample size of the convenience cross-sectional study was N = 317, with some participants completing the questionnaire a second time after 4 weeks, resulting in a subsample of n = 69. The present study followed the EQUATOR guidelines for reporting research using the “Strengthening the Reporting of Observational Studies in Epidemiology” (STROBE) checklist (Elm et al., 2007) (Supplementary Material S1).

### Questionnaire design

Google Forms were used to collect the data, which required participants to answer sequential questions, including questions about religiosity (three questions), previous experience with genetic testing, a self-assessment of how much the person knows about genetic technologies compared to the general population, Knowledge of Modern Genetics and Genomics (KMGG) with 16 questions adapted from Carver et al. (2017), awareness of genetic testing with 12 questions, adapted from Henneman et al. (2013), Chokoshvili et al. (2017), Chin and Tham (2020), followed by the 30 questions of the GTQ and five questions of the Conventional Technologies Questionnaire (CTQ) (Küchenhoff et al., 2022), which correspond to the short form of the GTQ, the CTQ5. At the end of the first questionnaire, participants were asked if they wished to participate a second time after 4 weeks and were given an option to create a code so that the data from the two surveys could be matched. Additionally, participants were asked to provide their email addresses so that they could be reminded to participate the second time. The time needed to complete the questionnaire was estimated to be around 10–20 min.

### Knowledge of Modern Genetics and Genomics (KMGG)

The KMGG is a part of the “Public Understanding and Attitudes towards Genetics and Genomics Questionnaire” developed by Carver et al. (2017). It measures an individual’s objective knowledge of genetics and genomics. The KMGG focuses on three areas: (1) characteristics of the genome, (2) gene function and expression, and (3) epigenetics. It consists of 16 statements that can be answered as “true,” “false,” or “I do not know.” The total score ranges from 0 to 16 points, with higher scores associated with better knowledge of genetics and genomics. The questionnaire achieved a Cronbach’s alpha of 0.69 and 0.70 in the pilot study. The German version of the KMMG questionnaire was validated in a parallel project and achieved a Cronbach’s alpha value of 0.85 (Melchior et al., 2024).

### Genetic Technologies Questionnaire (GTQ) and Conventional Technologies Questionnaire (CTQ)

The GTQ was developed by Küchenhoff et al. (2022) to assess the sensitivity of lay moral judgments to the moral status of the living being involved, data privacy concerns, and social justice issues. Human and non-human species were chosen to differentiate moral status, and genetic testing and editing were chosen to contrast the severity of the intervention. Thus, the questionnaire included items from six domains: human genetic testing, non-human genetic testing, human genome editing, non-human genome editing, privacy, and social justice. To avoid framing effects, items and response options were worded in a neutral way. The GTQ also exists as a 20-item questionnaire (GTQ20), which includes the items with the highest overall correlations with the total score, and as a five-item questionnaire (GTQ5), which covers only the topics of human and non-human genome editing. A contrastive design (Burstin et al., 1980) was used by developing an almost identical item for each item, replacing the expression about genetic technologies with an expression about conventional technologies (CTQ). In the current study, we did not use the 30-item CTQ but only the five items corresponding to the GTQ5. Ratings of moral goodness or badness were given on a 6-point Likert scale ranging from (1) “morally bad” to (6) “morally good” for both scales.

The GTQ30 managed to achieve an internal consistency of 0.95 in Küchenhoff’s original publication (Küchenhoff et al., 2022) and has been translated into Greek (Melchior et al., 2025) with excellent scale properties and an internal consistency of 0.93.

### Developing the German version of the questionnaires

The translation-back method (Hambleton, 2001) was used to translate the English version of the GTQ into German. Specifically, two native German speakers translated the original English version into German. Differences in translation were discussed with the research team, ensuring cultural adaptation and a synthesis of the two translations was produced. This German version was back-translated by a bilingual public health expert whose mother tongue was English and by an English translator. The original English version and the back-translated versions were compared for consistency, relevance, and meaning of the content. The German version was reviewed by five researchers with expertise in either genetics or ethics to ensure that all items were consistent before finalizing the German version of the questionnaires (GTQ-D). The German translation is provided in Supplementary Material S2.

### Statistical analysis

The data were analyzed using descriptive and inferential statistical methods with IBM SPSS Statistics Version 27 (IBM Corp, 2022). The psychometric properties of all questionnaires measuring a psychological construct were evaluated, including internal consistency (Cronbach’s alpha), structural validity (principal component analysis for the GTQ), construct validity (known-groups method), item analysis, floor and ceiling effects, and retest reliability.

### Internal consistency and retest reliability

To ensure internal consistency, we calculated Cronbach’s alpha. This measure captures the extent of shared variance among items and aids in evaluating the reliability of scales comprising more than 10 items. The recommended range for Cronbach’s alpha is 0.70–0.90 (Tavakol and Dennick, 2011). To assess retest reliability, we compared data from the full sample (N = 317) with the subsample (n = 69) after a 4-week interval and the intraclass correlation coefficient was calculated, which measures the similarity between the two surveys. The retest reliability was calculated according to Koo and Li (2016) in SPSS using a two-way mixed effects model with the mean of k measurements and absolute agreement. Due to the inclusion of incorrect email addresses, three individuals could not be identified and were subsequently excluded from the retest. Consequently, the retest was conducted with a total of 69 participants.

### Structural validity

To examine the factor structures, a principal component analysis (PCA) with Varimax rotation was performed on the GTQ30 and GTQ-H to explore the underlying factor structure and compare it with the original publication. The criteria for PCA were a Kaiser-Meyer-Olkin coefficient (KMO) greater than 0.6 and a significant Bartlett’s test of sphericity (Kaiser and Rice, 1974; Tabachnick and Fidell, 2014).

### Construct validity

To assess construct validity, we used the known-groups method, in which two groups are distinguished based on expected differences in their scale scores. Accordingly, separate known-groups analyses were conducted for the GTQ and CTQ, using age, gender, religiosity, and education as grouping variables. We formulated the following hypothesis for this study: (1) Older people score lower on the GTQ than younger people (young is defined as 18–30 years and old as 50+ years); (2) women score lower on the GTQ than men; (3) People with high religiosity score lower on the GTQ than people with low religiosity. To this end, it is asked how often participants attend church services, how religious they consider themselves to be, and how strongly their opinions are influenced by religion. The scale here ranges from 3 to 15, with 3 to 5 being considered less religious and 11–15 being considered more religious. The fourth hypothesis regarding the GTQ and CTQ states that individuals with higher levels of education will obtain higher scores on the GTQ compared to those with lower education levels. The study asks for the highest level of education attained, with “lower education” defined as no academic qualification and “higher education” with a bachelor’s degree or higher. These hypotheses were tested for all questionnaires using either the Wilcoxon-Mann-Whitney test (WMW) (Mann and Whitney, 1947) or the Kruskal–Wallis tests (Kruskal and Wallis, 1952).

### Power analysis

To ensure that our analyses have adequate statistical power, we conducted a power analysis using the GPower 3.1.9.7 software (Faul et al., 2007), following Kang’s guidelines (2021). The expected effect size for the hypotheses was at least d = 0.5, with a desired power of 1 - β = 0.95 and a significance level of α = 0.05. Given our previous studies (Teichmann et al., 2022; Melchior and Teichmann, 2023), which showed a slightly over-educated sample due to our recruitment approach, we set the allocation ratio to 3. As a result, we expected a higher percentage of individuals with advanced knowledge of genetic technologies in the sample, requiring a larger sample size. GPower recommended a total sample size of N = 244 for the Wilcoxon-Mann-Whitney tests.

### Item analysis

For each questionnaire, we conducted an item analysis to assess the item-total correlation of all items. This correlation measures the consistency between the score of an individual item and the total scale score and provides valuable insight into the explanatory power of each item. In addition, we examined the inter-item correlations to assess the strength of the relationships between items. In general, mean item-total and mean inter-item correlations of around 0.2 to 0.4 are considered to indicate a significant contribution of information to the scale. However, it is important to note that higher correlations do not necessarily imply higher reliability. In fact, excessively high correlations indicate item redundancy, which artificially inflates reliability (Ferketich, 1991; Rattray and Jones, 2007; Piedmont, 2014).

### Floor and ceiling effects

It is also essential to consider the presence of ceiling or floor effects as another criterion. A ceiling or floor refers to the maximum or minimum value that an observation can reach, such as a perfect score. When a variable accumulates at these extreme values, it creates a ceiling or floor effect, distorting the distribution and introducing bias that can generate misleading results in analyses that assume a normal distribution (Šimkovic and Träuble, 2019). Although specific thresholds for this effect are not universally defined, we considered it to be present if more than 10% of all participants scored at the minimum or maximum level on any questionnaire. In addition, we re-evaluated floor and ceiling effects specifically for the groups with low and advanced self-assessed knowledge of genetic technologies to determine whether the presence of a floor or ceiling effect was dependent on the specific sample.

There was no missing data, as Google Forms only accepted completed records.

### Pairwise comparisons of the GTQ domains

We performed a Friedman two-way analysis of variance with ranks, followed by a Dunn-Bonferroni *post hoc* test to test for pairwise differences to compare the eight different domains of the GTQ: (1) genetic testing on humans, (2) embryos, (3) plants, and (4) animals, and (5) genetic modification on humans, (6) embryos, (7) plants, and (8) animals (Friedman, 1937; Dinno, 2015). The Friedman test, a nonparametric ANOVA, assigns ranks to the ratings of the eight categories for each participant and assesses whether significant differences exist between these categories. The Dunn-Bonferroni test then identifies pairs with a significant difference and quantifies the magnitude of that difference using a z-value.

### Ethical considerations

This study was approved by the Ethics Committee of the Faculty of Behavioral and Empirical Cultural Sciences, Heidelberg University (AZ Teich 2022 3/1). All procedures involved in this work conformed to the ethical standards of the Declaration of Helsinki, as applicable to national and institutional human experimentation committees. Prior to the survey, we obtained written informed consent from each participant. The participants were informed that the research was voluntary, confidential, and for academic purposes only. After merging the data from different time points to ensure anonymity, the research team removed the email addresses from the server.

## Results

### Participants

A total of 317 people participated in the study, 69 of whom completed the questionnaire a second time after 4 weeks. Table 1 shows the sociodemographic data for both the total sample and the subgroup. The average age of the sample is 43.53 years and 47.22 years for the subgroup, with the majority of participants being female (69.7% and 68.1%, respectively). The most common level of education attained is a Master’s degree or diploma, accounting for 38.8% of the total sample and 40.3% of the subgroup consisting of participants primarily working in academic positions (28.1% of the total sample and 22.2% of the subgroup).

**TABLE 1:** Participant characteristics of the total sample and the subgroup.

Characteristics	Full sample (N = 317)		Subgroup^a^ (n = 69)	
	*n*	*%*	*n*	*%*
Age				
Mean	43.53		47.22	
SD	17.87		18.95	
Gender				
Male	93	23.3	20	29.0
Female	221	69.7	47	68.1
Diverse	3	0.9	2	2.9
Education				
9 years or less	0	0.0	0	0.0
10 years	7	2.2	3	4.4
12-13 years	70	22.1	12	17.4
Vocational training	35	11.0	7	10.1
Bachelor	36	11.4	9	13.0
Master/Diploma	123	38.8	28	40.6
PhD	44	13.9	10	14.5
Others	2	0.6	0	0.0
Occupation				
School student	2	0.6	1	1.5
Student	78	24.6	16	23.2
Unemployed	3	0.9	0	0.0
Retiree	37	11.7	15	21.7
Care profession	11	3.5	3	4.4
Therapeutical profession	21	6.6	2	2.9
Physician	12	3.8	3	4.3
Academic	100	28.1	15	21.7
Others	53	20.2	14	20.3
Marital status				
Divorced	20	6.3	4	5.8
In partnership	81	25.6	14	20.3
Single	84	26.5	16	23.2
Married	126	39.7	33	47.8
Widowed or deceased partner	6	1.9	2	2.9
Do you have children?				
yes	142	44.8	37	53.6
no	175	55.2	32	46.4
Have you ever had a genetic test done?				
yes	20	6.3	6	8.7
no	297	93.7	63	91.3
Has genetic testing ever been performed on a close friend or relative?				
yes	72	22.7	11	16.0
no	128	40.4	33	47.8
I don’t know	117	36.9	25	36.2
Would you like to have a genetic test performed?				
yes	78	24.6	13	18.8
no	103	32.5	22	31.9
I don’t know	136	42.9	34	49.3

^a^ Subgroup after four weeks for the retest.

### Sociodemographic data

When asked about genetic testing, 6.3% of the total sample and 8.3% of the subgroup reported having undergone genetic testing. In addition, 22.7% of the total sample and 15.3% of the subgroup have had a genetic testing done on a close friend or relative. While 24.6% of the total sample and 19.4% of the subgroup expressed a desire to undergo genetic testing, 32.5% of the total sample and 30.6% of the subgroup did not.

Religiosity was assessed by three questions about self-rated religiosity, frequency of attending religious services, and how religion influences decisions, which were summed. The mean score was M = 5.79 (SD = 2.77) on a scale of 3–15. When we categorised the variable as high (11–15), medium (6–10) and low (3–5), we found that the majority of individuals (n = 176) scored low on religiosity. Meanwhile, 116 people scored medium and only 25 people scored high.

In the self-assessment of how much a participant knows about gene technologies compared to the general population, a mean value of M = 4.31 (SD = 1.38) was achieved on a scale of 1–7. Most people fall into the “medium” (3–5 points, n = 140) and “high” (6–7 points, n = 141) categories, with only 36 people rating their knowledge of genetic technologies as low (1–2 points). When participants were asked which genes they had been tested for, the most commonly mentioned were those associated with breast cancer, ovarian cancer, general cancer susceptibility, Down syndrome (trisomy 21), and health screening. The motivations for these tests often included familial reasons, such as having affected family members or close relatives, as well as future planning, early detection, and curiosity.

The participants who expressed a future interest in undergoing genetic testing were asked to provide a rationale for their motivations. The most frequently cited motivations included cancer, dementia, Parkinson’s disease, family history, prevention, and early detection. Additionally, a number of participants indicated a willingness to participate in testing for all potential conditions, motivated not by specific reasons but rather by a general interest in the subject.

## Genetic Technologies Questionnaire (GTQ)

### Correlations

Table 2 provides an overview of the correlations between the GTQ30, other GTQ variants, and the variables considered in this dataset. All presented correlations were analyzed using Spearman’s rank correlation.

**TABLE 2:** Correlations between the scales and other variables.

Item/Scale	2	3	4	5	6	7	8	9	10	11	12	13	14	15
1. GTQ30	0.981**	0.899**	0.771**	0.918**	0.939**	0.733**	−0.225**	0.044	−0.232**	0.113*	0.119*	0.496**	0.478**	−0.254**
2. GTQ20		0.927**	0.798**	0.865**	0.929**	0.646**	−0.232**	0.058	−0.213**	0.116*	0.132*	0.460**	0.452**	−0.251**
3. GTQ5			0.788**	0.816**	0.809**	0.541**	−0.269**	0.035	−0.165**	0.032	0.090	0.415**	0.421**	−0.229**
4. CTQ5				0.643**	0.748**	0.491**	−0.177**	0.144*	−0.097	0.151**	0.195**	0.328**	0.367**	−0.279**
5. GTQ-H					0.826**	0.788**	−0.270**	0.015	−0.245**	0.059	0.056	0.500**	0.474**	−0.187**
6. GTQ-MS						0.727**	−0.151**	0.084	−0.217**	0.174**	0.137*	0.464**	0.429**	−0.280**
7. Human Testing^a^							−0.202**	0.005	−0.269**	0.100	0.090	0.489**	0.436**	−0.205**
8. Age								0.107	0.216**	−0.050	0.037	−0.206**	−0.183**	−0.070
9. Years of Education									0.097	0.288**	0.253**	0.001	−0.009	−0.008
10. Religion										−0.038	0.042	−0.075	−0.106	0.002
11. KMGG^b^											0.475**	0.025	0.062	−0.069
12. SAK^c^												0.080	0.058	−0.199**
13. Genetic test^d^													0.763**	−0.164*
14. Profiling test^e^														−0.145*
15. Gender^f^														

^a^ Items 1, 2, 3, 4, 21, which include statements about genetic testing for humans.

^b^ Knowledge of Modern Genetics and Genomics questionnaire.

^c^ Self-assessed knowledge about genetic technologies.

^d^ Would you like to have a genetic test performed? (0 = no, 1 = yes, “I do not know” was excluded).

^e^ I would take a test to create a genetic profile to find out if I am at risk of developing certain diseases (1 = strongly disagree to 5 = strongly agree).

^f^ male = 0, female = 1.

*Significant at the level p < 0.05; **significant at the level p < 0.001.

### Descriptive statistics

The descriptive statistics for the GTQ30 and all other questionnaires are presented in Table 3. The GTQ30 achieved a mean of 3.347 (SD = 0.849) on a scale from 1 to 6. The sum of the total score is divided by the number of items, which means that all GTQ versions have the same range. The mean score for the GTQ20 (M = 3.339; SD = 1.011) was nearly equal to the GTQ30, and the GTQ5 had a slightly lower mean (M = 2.976; SD = 1.226).

**TABLE 3:** Psychometric properties of all tested scales.

Scale	Mean score (SD) [range]		Cronbach’s alpha		Mean item-total correlation	Mean inter-item correlation
	Total sample	Total sample	LK^a^	HK^b^	Total sample	Total sample
GTQ30^c^	3.347 (0.849) [1,6]	0.938	0.955	0.927	0.556	0.330
GTQ20	3.339 (1.011) [1,6]	0.940	0.954	0.932	0.641	0.437
GTQ5	2.976 (1.226) [1,6]	0.857	0.886	0.853	0.672	0.546
GTQ-H	3.219 (0.792) [1,6]	0.881	0.917	0.856	0.517	0.303
GTQ-MS	3.875 (0.966) [1,6]	0.779	0.853	0.779	0.484	0.304
CTQ5^d^	3.771 (0.964) [1,6]	0.697	0.820	0.582	0.457	0.314

^a^ LK, low self-assessed knowledge about genetic technologies.

^b^ HK, high self-assessed knowledge about genetic technologies.

^c^ Genetic Technologies Questionnaire.

^d^ Conventional Technologies Questionnaire.

### Internal consistency and retest reliability

Internal consistency scores are shown in Table 3 for the total sample and separately for the two self-assessed knowledge groups. The Cronbach’s alpha values for GTQ30, GTQ20, and GTQ5 are excellent (0.938, 0.940, 0.857, respectively), with the values for GTQ30 and GTQ20 exceeding the recommended threshold of 0.9. In the two groups with high and low self-assessed knowledge of genetic technologies, the values of internal consistency remain constant and do not show any sample-dependent weakness.

Table 4 presents the results of the retest, showing the intraclass correlation coefficient along with its 95% confidence intervals. Notably, all GTQ variants showed commendable performance during the retest. The GTQ30 scored the highest at 0.929, closely followed by the GTQ20 at 0.908 and the GTQ5 at 0.879.

**TABLE 4:** Test-retest reliability with the subgroup (n = 69).

Scale	Intraclass correlation coefficient	95% - confidence interval	
		Lower bound	Upper bound
GTQ30	0.929	0.886	0.956
GTQ20	0.908	0.852	0.943
GTQ5	0.879	0.804	0.925
GTQ-H	0.935	0.894	0.961
GTQ-MS	0.884	0.810	0.929
CTQ5	0.840	0.742	0.901

### Structural validity

The criteria for PCA were met with a KMO of 0.925 and a significant Bartlett’s test for sphericity χ^2^ (435) = 5,229.61, p < 0.001. The PCA for the GTQ30 revealed five factors. The first factor consists of items 30, 28, 25, 8, 26, 29, 27, 7, 23, and 24 (sorted in descending order of factor loading) and deals with genetic modification of animals and plants. The second factor (items 22, 18, 19, 15, 16, and 17) deals with disease prevention and cognitive enhancement. Factor 3 (items 2, 3, 4, 21, 12, 20, and 1) includes items related to determining the risk of genetic diseases. The fourth factor includes items related to data protection (9, 11, 10, and 13), and the fifth factor contains only one item, namely item 14: “Mitigating punishment based on the genetic predisposition of the offender is ․․․”. The remaining items 5 and 6 were grouped into a separate factor, which, however, has a large overlap with factor 1 and includes genetic testing on animals.

### Construct validity

The results of the known-groups method for gender, education, age, and religion are presented in Table 5. All variants of the GTQ were effective in distinguishing between male and female participants, in making a significant distinction between older and younger participants, and in identifying individuals from high and low religious affiliation groups. However, when the groups of academic and non-academic participants were examined, none of the GTQ variants showed differences in test scores. Unfortunately, the group of diverse participants was too small to yield statistically significant results.

**TABLE 5:** Known-groups analysis for all questionnaires.

Group (n)	Gender		Education		Age		Religion		
	Female (221)	Male (93)	Non-academic (112)	Academic (202)	Low (106)	High (128)	Low (176)	High (25)	Significance
	Mean rank								
GTQ30	141.48	197.81	165.47	153.08	190.43	142.79	107.21	57.30	Gender** Age** Religion**
GTQ20	141.70	197.34	164.46	153.64	190.71	142.48	107.29	56.70	Gender** Age** Religion**
GTQ5	142.94	194.25	166.52	152.50	195.18	139.39	107.39	56.04	Gender** Age** Religion**
GTQ-H	145.60	188.05	168.26	151.53	195.01	138.61	107.58	54.66	Gender** Age** Religion**
GTQ-MS	141.06	200.41	159.31	156.50	181.62	148.32	106.57	61.78	Gender** Age* Religion**
CTQ5	139.69	201.62	158.81	156.77	180.84	146.08	104.81	74.16	Gender** Age* Religion*

Annotations: WMW tests were used for education and religion, Bonferroni corrected Kruskal–Wallis tests were used for gender and age.

**Significant at the level p < 0.001. *Significant at the level p < 0.05.

### Item analysis

The item analysis of the GTQ is mostly satisfactory; however, it becomes apparent that there are some items that show excessive similarity. While this can be seen by comparing the content of the items, this suspicion can be confirmed by examining the inter-item and item-total correlations. With a mean item-total correlation of 0.556, the GTQ30 is above the recommended threshold of 0.4. This is also true for the GTQ20 and GTQ5, which have even higher mean correlations of 0.641 and 0.672, respectively.

The examination of individual items revealed that there are some redundant statements that add little new information to the questionnaire because they are already covered by other items. For example, item 30 “The genetic modification of plants to improve seed quality is ․․․” and item 28 “The genetic modification of seeds to improve nutritional value is ․․․” have a correlation of 0.842, which makes them statistically almost identical. Other high inter-item correlations are observed between items 8 and 24 to 30.

**FIGURE 1:**
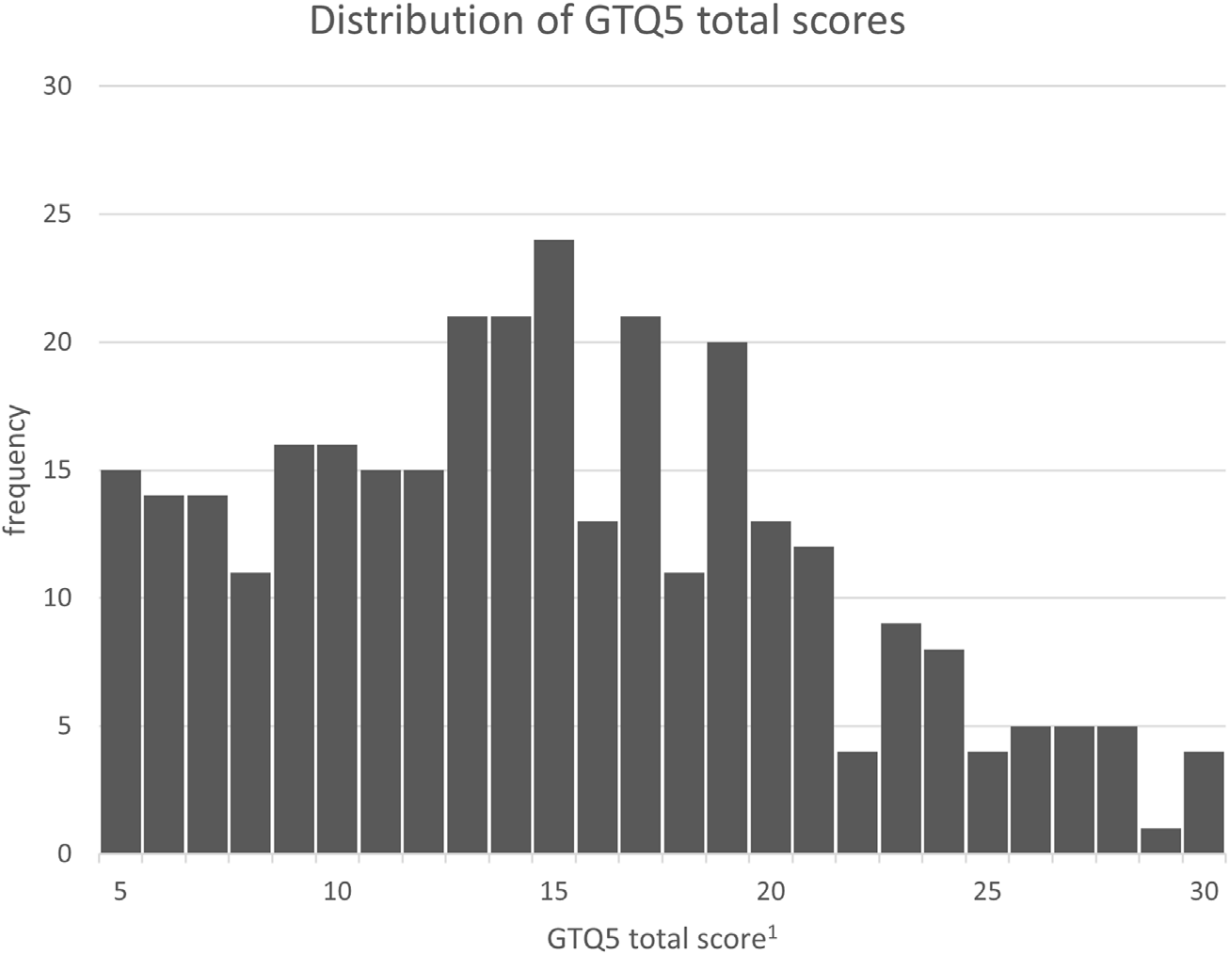
Distribution of GTQ5 total scores. ^1^GTQ5 raw total score on a scale from 5 to 30. This score is later divided by the number of items, which results in a final scale from 1 to 6.

Neither the GTQ30 nor the GTQ20 show evidence of floor or ceiling effects. Only two individuals scored at the maximum, and no individual scored at the minimum. For the GTQ5, however, there is an observable clustering of scores at the lower end of the scale. The distribution of the GTQ5 has a skewness of 0.351 with a standard error of 0.137, resulting in a z-value for skewness of 2.56, indicating a significant deviation from the normal distribution. The distribution is shown in Figure 1 for better understanding.

### Interest in and experience with genetic testing

In addition, we examined the possible influence of three variables present in the sociodemographic data on the GTQ30: 1) whether an individual had undergone genetic testing, 2) whether a close friend or relative had undergone genetic testing, and 3) whether the participant expressed interest in genetic testing. The WMW test examined whether these groups differed in their GTQ30 scores, and the results are shown in Table 6.

**TABLE 6:** Results of the Wilcoxon-Mann-Whitney tests for interest in and experience with genetic technologies.

Item	Mean rank		U^a^	z^b^	p^c^
	Yes (n = 72)	No (n = 128)			
Has genetic testing ever been performed on a close friend or relative?	108.55	95.97	4028.5	1.475	0.140
	Yes (n = 78)	No (n = 103)			
Would you like to have a genetic test performed?	120.79	68.44	7049.0	6.659	<0.001
	Yes (n = 20)	No (n = 297)			
Have you ever had a genetic test done?	193.73	156.66	2275.5	1.751	0.080

^a^ U = U test statistic.

^b^ z = z statistic.

^c^ p = significance, a higher mean rank is associated with a higher mean GTQ30 score.

Individuals who have a close friend or relative who has undergone genetic testing do not show significantly different responses on the GTQ items. Similarly, individuals who have undergone genetic testing themselves do not differ in their GTQ scores from those who have not undergone genetic testing. However, there is a notable difference between those who have expressed a willingness to undergo genetic testing and those who have not. Those who have expressed interest in genetic testing have significantly higher GTQ scores (p < 0.001, z = 6.659).

We also divided the participants into three groups: (1) want to undergo genetic testing, (2) do not want to undergo genetic testing, and (3) are unsure if they want to undergo genetic testing. In this context, it was checked whether this attitude was also reflected in the item “I would take a test to create a genetic profile to find out if I am at risk of developing certain diseases” from the Awareness of Genetic Testing Questionnaire. The results are shown in Table 7.

**TABLE 7:** Consistency in the interest for genetic testing.

	I would take a test to create a genetic profile to find out if I am at risk of developing certain diseases				
Would you like to have a genetic test done?	Strongly disagree	Disagree	Neutral	Agree	Strongly agree
yes (n = 78)	0	2	2	37	37
no (n = 103)	21	48	14	17	3
I don't know (n = 136)	5	34	32	45	20

Note. Of the participants who initially indicated an interest in genetic testing, almost all confirmed this interest in the follow-up question about genetic testing. Of the 103 participants who expressed aversion to genetic testing, 20 changed their minds and indicated that they were interested in their genetic profile. Similarly, of the 136 undecided participants, a total of 65 expressed interest in genetic testing in the follow-up question.

### Moral judgment of the different domains

**FIGURE 2:**
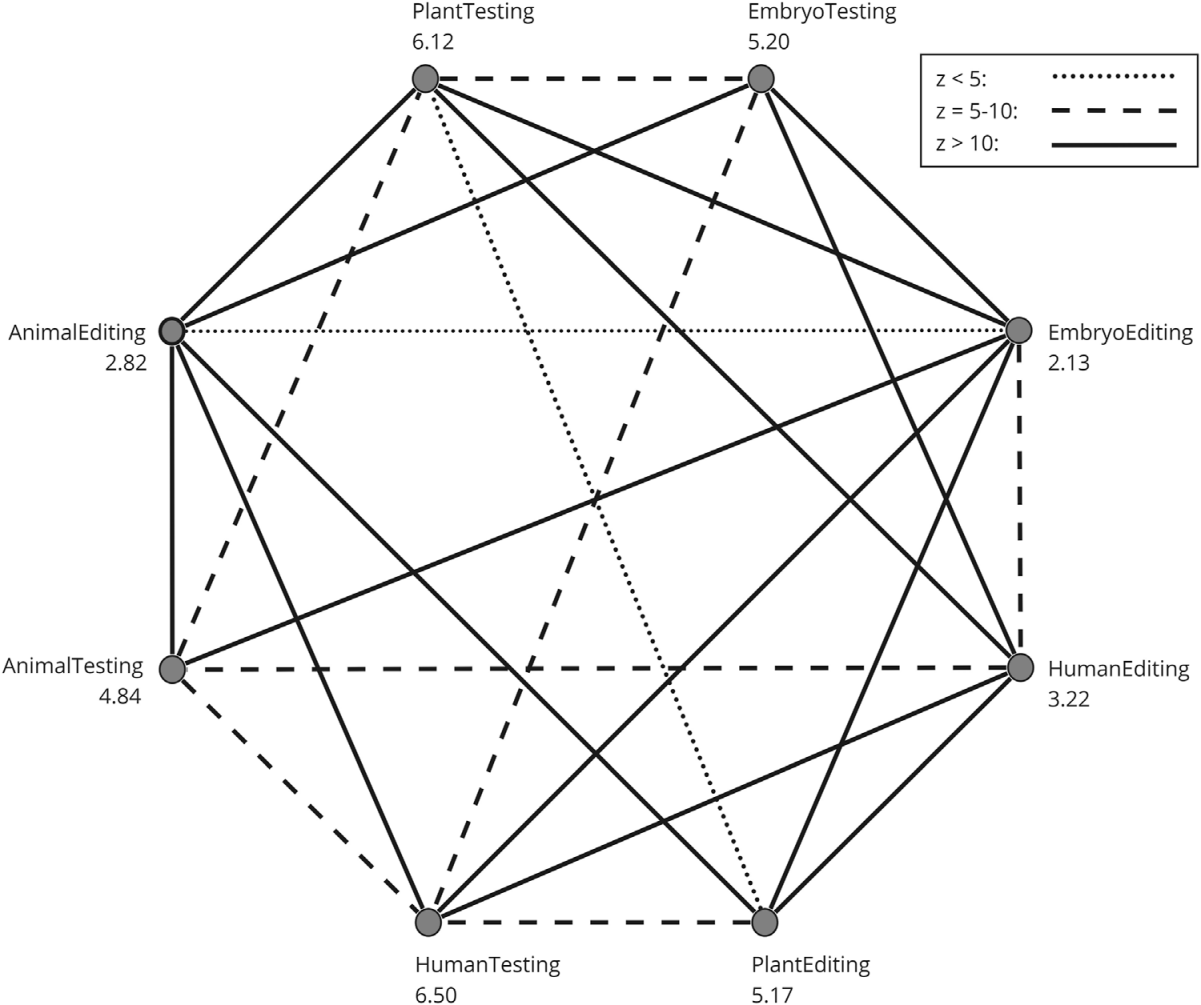
Post-hoc pairwise comparison of the GTQ domains. The numbers on the eight categories are the mean ranks from the Friedman test. Only pairs with a significant difference are connected with a line. The type of line is determined by the z-value of the pairwise difference.

To assess how participants evaluate the eight different areas: genetic testing on (1) humans, (2) embryos, (3) plants, and (4) animals, as well as genetic modification on (5) humans, (6) embryos, (7) plants, and (8) animals, a Friedman’s two-way analysis of variance by ranks was conducted, followed by a Dunn-Bonferroni *post hoc* test to examine pairwise differences. Figure 2 presents a chart where each pairwise comparison was examined for significance. All pairs that exhibit a significant difference are connected by a line, and the type of line represents the z-value of that difference. The numbers on the eight categories represent the average ranks from the Friedman test, ranging from 1 to 8. Several key insights emerge from this analysis: Genetic testing is consistently rated as significantly more favorable morally compared to its genetic editing counterparts, such as animal testing and animal editing. The moral rating for genetic modification in embryos is the lowest, whereas genetic testing in adults receives the highest moral rating. Meanwhile, the moral ratings for gene editing in animals and adults are similar and significantly lower than genetic modification in plants.

### GTQ-H and GTQ-MS

In addition to the GTQ30 and its shortened versions, two other categories related to the moral evaluation of genetic technologies were of interest, which are already covered by the items of the GTQ30. These are the GTQ-Human (GTQ-H), which contains only human-related items (items 1, 2, 3, 4, 9, 10, 11, 12, 13, 15, 16, 17, 18, 19, 20, 21, 22), and the GTQ-Moral Status (GTQ-MS), which includes moral status items (items 1, 2, 6, 7, 17, 20, 29, 30). The psychometric properties of both questionnaires are shown in Tables 2–5.

The GTQ-H has a good internal consistency with a Cronbach’s alpha of 0.881, and the GTQ-MS has a lower alpha of 0.779, which is expected since all items cover different factors and no similar items are used for the same topic.

The mean item-total correlation for the GTQ-H at 0.517 is similar to other GTQ variants, and the GTQ-MS has the lowest item-total correlation at 0.484. Due to the absence of high-correlation items related to plants in these two GTQ variants, the average inter-item correlation drops to 0.303 for the GTQ-H and 0.304 for the GTQ-MS, which is an optimal value.

The test-retest reliability for the GTQ-H was very good at 0.935 (95% CI: 0.894–0.961), and slightly lower for the GTQ-MS at 0.884 (95% CI: 0.810–0.929).

The principal component analysis (PCA) of the GTQ-H clearly identified three factors: Genetic Modification (Items 18, 19, 22, 15, 16, 17; sorted by factor loading), Genetic Testing (Items 2, 4, 3, 12, 21, 1, 20), and Data Privacy (Items 11, 9, 10, 13). The factor loadings for the GTQ-H are included in the Supplementary Material S3. The three factors of the GTQ-H have the following internal consistencies: first factor (Genetic Modification): 0.866, second factor (Genetic Testing): 0.807 and third factor (Data Privacy): 0.650. In the known-groups analysis, the GTQ-H and GTQ-MS were able to distinguish between male and female participants, younger and older participants, as well as individuals with low and high religiosity, and both failed to detect significant differences in academic and non-academic groups.

Neither the GTQ-H nor the GTQ-MS showed evidence of ceiling or floor effects.

## CTQ5

### Descriptive statistics

The mean score of the CTQ was 3.77 (SD = 0.946) on a scale of 1–6. Since the CTQ5 was derived from the GTQ5 through a contrastive design, it was of interest to examine how these two questionnaires differed from each other. Therefore, both questionnaires were compared using a paired samples Wilcoxon signed rank test. The results indicate that the CTQ5 scores (Mdn = 3.8, IQR = 1.60) are significantly higher than those of the GTQ5 (Mdn = 3.0, IQR = 1.80) (z = 13.526, p < 0.001).

In addition, we examined whether religious individuals perceive the statements of the CTQ5 as more moral than those of the GTQ5. For this purpose, we compared the CTQ5 and GTQ5 scale scores within the same individual using a Wilcoxon signed-rank test. It turns out that a person with high religiosity has a significantly higher total score on the CTQ5 compared to the GTQ5 (CTQ5 Mdn = 3.4, IQR = 1.3; GTQ5 Mdn = 2.0, IQR = 1.6; z = 4.294, p < 0.001).

### Internal consistency and retest reliability

In the total sample, the CTQ5 achieved a relatively low Cronbach’s alpha value of 0.697, which dropped to 0.582 in the group with high knowledge of genetic technologies. The reason for this is likely that the item “Changing the hormone balance of farm animals to reduce costs without harming them is … ” is rated very negatively (M = 2.52, SD = 1.440), while on the other hand, the item “Vaccinating human adults to protect them against influenza is … ” is rated very positively (M = 5.32, SD = 1.038). The resulting divergence between these two items leads to a significant reduction in their shared variance and causes a decrease in the internal consistency of the questionnaire. The retest is satisfactory, with a confidence interval of 0.742–0.901.

### Construct validity

Construct validity results are almost identical to those of the GTQ versions. The CTQ5 was able to successfully discriminate between genders, young and old individuals, and non-religious and religious individuals. However, one difference from the GTQ versions is that in the case of the CTQ5, the difference between the non-religious and religious groups was not as significant, as can be seen from the difference in mean ranks.

### Item analysis

In the item analysis, the CTQ5 achieved a mean item-total correlation of 0.457 and a mean inter-item correlation of 0.314. Both values are within the desired range, although the former slightly exceeds it. However, it is worth noting that item 3 “Changing the hormones of farm animals to improve their wellbeing is …” and item 4 “Changing the hormone balance of farm animals to reduce costs without harming them is ․․․” have a correlation of 0.70, indicating high redundancy between the two items.

### Floor and ceiling effects

Less than 1% of individuals were able to achieve the minimum and maximum scores on the CTQ5, indicating no distributional bias as observed with the GTQ5.

### Comparison to the original study

Because Küchenhoff et al. (2022) examined a comprehensive set of hypotheses in their study and presented the results in tabular form, it is advisable to examine these with our sample as well and compare our results with the original study, which was conducted with a representative sample of the U.S. population.

Table 8 lists the hypotheses formulated and tested by Küchenhoff et al. (2022) that can also be analyzed with our dataset. The table indicates whether each hypothesis can be confirmed in this sample or not, and whether it was confirmed in the original study.

**TABLE 8:** Comparison with Küchenhoff et al. (2022).

No.	Statement	This sample	Küchenhoff et al. (2022)
1	Genetic editing of human adults is regarded as better than that of embryos (the mean rating of GTQ18^a^ is greater than that of GTQ19, that of GTQ15 is greater than that of GTQ22)	✔	✔
2	Overall, ratings of genetic testing (GTQ1-8) correlate with ratings of genome editing (items GTQ15-30)	✔	✔
3	Overall, ratings of genome editing are lower (morally worse) than of genetic testing (the mean rating of GTQ15-30 is lower than that of GTQ1-8)	✔	✔
4	Participants self-identified as male rate the use of genetic technologies on animals (items GTQ5, 6, 23, 24, 27, 29) as morally better than participants self-identified as female	✔	N/A
5	Participants rate genetic technologies as morally better when they are used to improve nutritional value (GTQ28) or fight world poverty (GTQ25) than to improve taste (GTQ26)	✔	✔
6	Participants rate genetic technologies as morally better when they are used to improve wellbeing (GTQ23) rather than to increase efficiency (GTQ6)	✘	✘
7	Genome editing of embryos is rated as morally better when performed in order to prevent a fatal disease (GTQ17) than when used to prevent influenza (GTQ19)	✔	✔
8	Genome editing of human adults is rated as morally better when performed in order to treat cancer (GTQ20) than when used to protect them against influenza (GTQ18)	✔	✘
9	The higher the participant’s education (measure of education level, years of education) the higher the GTQ total score	✘	✔
10	The more religious participants consider themselves to be, the worse they rate genome editing (GTQ15-30)	✔	✘
11	Participants who already had experience with genetic tests rate genetic technologies as morally better (GTQ total score)	✘	✘
12	The more participants think they know about genetic technologies, the more extreme (trending away from the midpoint of the scale) they rate the morality of genetic technologies (positive or negative)	✘	✘
13	Objective knowledge about genetics is negatively correlated with opposition to genetic technologies	✔	✔
14	A discrepancy between self-assessed and objective knowledge about genetic technologies is positively correlated with opposition to genetic technologies	✘	✘

^a^ GTQ18 refers to item 18 from the GTQ, and so do all the other GTQXs in this table.

When examining the results, it becomes clear that there are some differences compared to the sample of Küchenhoff et al. (2022). However, for most hypotheses we come to the same conclusions as they did. For the first statement, Wilcoxon signed-rank tests also revealed that genetic modification in adults is perceived as significantly more morally acceptable than genetic modification in embryos (GTQ18 and GTQ19: z = 7.634, p < 0.001; GTQ15 and GTQ22: z = 3.972, p < 0.001). The correlation between genetic testing (items 1–8) and genetic modification (items 15–22) was confirmed to be ρ = 0.698 (p < 0.001).

The Wilcoxon signed-rank test confirmed that genetic testing was morally superior to genetic modification (z = 15.172, p < 0.001). Genetic testing had a mean of 4.420 (SD = 0.902), while genetic modification had a mean of 3.153 (SD = 1.056).

We hypothesized that male participants would rate the use of genetic technologies on animals as morally better than female participants. This hypothesis was confirmed as male participants achieved an average rank of 201.24 and female participants 139.02 in the WMW test (z = 5.544, p < 0.001).

Improving nutrition (z = 11.343, p < 0.001) and fighting poverty (z = 13.251, p > 0.001) are perceived as more morally acceptable than improving taste. In addition, the sixth hypothesis was also rejected in this sample (z = 0.896, p = 0.370). Statement seven was confirmed with z = 10.780 and p < 0.001. However, in contrast to Küchenhoff et al. (2022), we observed in our sample that genetic modification for cancer treatment (GTQ20) was rated as morally superior to genetic modification for protection against influenza (GTQ18) with z = 7.761, p < 0.001.

Our sample showed no significant correlation between the highest level of education and the GTQ score (ρ = −0.101, p = 0.074), nor between the number of years of education and the GTQ score (ρ = 0.044, p = 0.431). However, in this sample, we were able to demonstrate a negative correlation between religiosity and the evaluation of genetic technologies: ρ = −0.216, p < 0.001.

Hypothesis 11could not be confirmed (z = 1.751, p = 0.080), and in hypothesis 12, no relationship was found between self-rated knowledge of genetic technologies and the difference from the scale mean of the GTQ30 (ρ = −0.028, p = 0.615). However, it is worth noting that individuals who rated their knowledge as maximum (7 on a scale of 1–7) showed the greatest difference from the mean.

There was a slight positive correlation between the KMGG score and the GTQ30 score (ρ = 0.094, p = 0.048). For the final hypothesis, we examined the discrepancy between self-rated knowledge and objective knowledge (KMGG total score) using normalized scale scores. However, the hypothesis could not be confirmed as this discrepancy did not correlate with the GTQ30 total score (p = 0.407).

## Discussion

### Scale properties

The aim of the present study was to translate and validate the GTQ with the German population. The GTQ showed excellent internal validity in all variants (GTQ30, GTQ20, GTQ5) and good to excellent reliability over time. PCA revealed a coherent five-factor structure except for item 14, forming a separate factor. One feature of the GTQ that should be highlighted is the high correlation between items. Although this is not a critical issue in itself, it does mean that the questionnaire is longer than necessary and that both the completion and scoring of the items take considerably more time. While no ceiling or floor effects were observed in the GTQ30 and GTQ20, the GTQ5 suffers from a clustering of test scores at the lower end of the scale, resulting in a floor effect and a notable weakness of the questionnaire. The CTQ5 also has strong properties, but its internal consistency is compromised by the fact that one item is rated exceptionally positively while another is rated exceptionally negatively. If future projects show that the item “Changing the hormone balance of farm animals to reduce costs without harming them is ․․․” consistently receives very low ratings, it may be advisable to consider removing it or using a longer version than the CTQ5 to mitigate the effect of this discrepancy.

As hypothesized, our findings indicate that conventional technologies were judged to be morally better than genetic technologies, and genetic testing was considered to be more favorable than genome editing. Furthermore, genome editing of embryos and humans was judged better when done to prevent or treat a serious disease than when done to prevent influenza, in addition, human genome editing was judged worse than non-human editing, while animal genome editing was considered to be worse than plant editing. Less religious people, men, younger people, and those interested in genetic testing rated genetic technologies higher on the Likert scale, which means that they consider them to be morally better.

The second aim of the study was to develop a comprehensive scale – the GTQ-H – that includes all human-related items. In addition, the GTQ-MS was developed, a scale that includes an item for genetic testing and an item for genome editing for each moral status. Both scales are reliable and valid tools for use in research.

### GTQ-H and its relevance for ethics

The GTQ was developed as a tool for experimental research and applied ethics, identifying ethically relevant issues from the philosophical literature. It aimed to address many different areas, i.e. to contrast genetic testing with genome editing, to include different reasons for testing and genome editing that have different values in terms of their benefits (e.g., protection against influenza versus protection against serious disease or improvement of taste versus alleviation of poverty). At the same time, the objective of the study was to consider the moral status of different living beings, as well as issues of autonomy and data security/privacy, and justice distribution. In this way, the four moral principles of Beauchamps and Childress (2019) should be taken into account: autonomy, beneficence, non-maleficence, and justice.

Since the moral acceptability of human application is of particular interest for most ethical research questions in medicine and biotechnology, we decided to combine all human-related items into one scale and analyse their psychometric properties. As reported earlier, the GTQ-H has a good internal consistency, with optimal inter-item correlations and a very good test-retest reliability. In addition, a clear three-factor structure was identified for the scale, which divides the GTQ-H into three thematic areas: genetic modification, genetic testing, and privacy. Since item 14 did not load onto any of these factors, we omitted this item from the compilation of the scale, even though it belongs to the human domain. While the first two factors show good internal consistency, we would recommend adding another item to the third factor that strengthens the construct. This scale seems to be an appropriate tool for experimental research to investigate judgments on ethical issues related to human genetic technologies. While there are numerous studies on public attitudes toward gene therapy or genome editing (McCaughey et al., 2016; Wang et al., 2017; Delhove et al., 2020), there is a scarcity of research focusing on moral judgments, possibly due to the lack of an appropriate tool.

### GTQ-MS and its relevance for ethics

In order to examine the moral judgments of different groups of living beings (plants, animals, human embryos, and adults), we have combined those items of the questionnaire that cover the four different groups of living beings - each with respect to genetic testing and genome editing - into the GTQ-MS. The questionnaire has excellent internal consistency, with no excessive correlations between items. Again, on this scale, men, younger people, and less religious individuals tend to be more morally supportive of genetic testing and genome editing compared to women, older people, and religious people. As expected, genetic testing was rated as morally better than genome editing, regardless of the species studied. Also, genetic testing and genome editing were seen as more morally acceptable for adults than for embryos and for plants than for animals.

This replicates the findings of Küchenhoff et al. (2022), who showed that moral judgments are sensitive to moral status as well as to the severity of the reason. Similar results were reported by Gaskell et al. (2017), who demonstrated that adult enhancement and prenatal therapy appear to be morally ambiguous. Küchenhoff et al. (2022) did not provide a detailed report of their findings regarding moral status, including differences between using animals or plants for research or to produce food or transplantable organs for humans. However, we believe that this scale would be beneficial for further research on lay perceptions of the moral status of different living beings as well as for studies relating to the use of model organisms in justifying/determining whether and what types of animals should be used in research or in answering the question whether experiments with animals can be justified to produce benefits to humanity (Neuhaus, 2018).

### CTQ *versus* GTQ

The use of a contrastive design to contrast genetic technology with conventional technology is a promising approach. In order not to overwhelm the participants with the length of the questionnaire, we included only the short form of the CTQ with five items corresponding to the GTQ. However, we were able to clearly demonstrate, as Küchenhoff et al. (2022) did, that conventional technologies are judged to be morally superior to genetic technologies. The GTQ5 takes moral status into account by including questions about embryos, adults, animals, and plants, but only items about genome editing are considered. It is, therefore, not surprising that the mean score is significantly lower than that of the GTQ30, which also includes questions about genetic testing and privacy, which are generally rated as morally superior.

### Moral judgment of genetic technologies

Most of the results of our study in the German population are consistent with those of the representative sample of the U.S. population that participated in the original study by Küchenhoff et al. (2022), as well as with studies examining attitudes toward genetic technologies (Delhove et al., 2020). For example, genome editing of human adults is regarded as being more widely accepted than in embryos (Gaskell et al., 2017). This is no longer a hypothetical question, as in December 2023 Casgevy, the first CRISPR/Cas9-based gene therapy for sickle cell disease (SCD), was approved by the FDA for treating patients aged 12 years and older with recurrent vaso-occlusive crises (VOCs). The potential benefits of Casgevy have sparked a broad debate about social justice and distributive justice, particularly given the significant costs associated with its introduction (Singh et al., 2024). Gene therapy has the potential to treat more than 10,000 monogenic human diseases and an even larger number of complex polygenic diseases (Memi et al., 2018), with monogenetic diseases and diseases affecting only single organs or tissues showing the most promise (Koch and Koster, 2021).

However, most countries allow gene therapy only to be performed on somatic cells, while the legal norms on germline interventions in international and supranational law vary (Nordberg and Antunes, 2022). Nonetheless, despite the progress, also somatic gene therapy is still not without risks, such as undirected integration of donor DNA at inappropriate sites, which can disrupt the function of previously intact genes (Silva-Pilipich et al., 2023).

The Biomedicine Convention of the Council of Europe (Art. 13) (Conseil de l'Europe, 1997) generally prohibits any intervention “intended to modify the genome of the offspring.” However, it has not yet been ratified by a number of states, including the Federal Republic of Germany, Belgium, and Ireland. The UNESCO Universal Declaration on the Human Genome and Human Rights (Art. 24) (UNESCO, 1997) states that interventions in the human germ line “may be contrary to human dignity.” According to Art. 3 (2b) of the EU Charter of Fundamental Rights (*Charter of Fundamental Rights: Home Page*, 2023), eugenic practices are generally inadmissible. This may also apply to germ-line interventions, although therapeutic applications could be exempted. The current legal framework regarding human genome editing is complex and requires revision due to continual scientific progress (Nordberg and Antunes, 2022). The only agreement reached so far is that neither germ-line (Albrecht et al., 2021) nor embryo-based therapies are allowed (Cyranoski and Reardon, 2015).

In their systematic review, Delhove et al. (2020) examined 41 studies on gene therapy and genome editing for acceptability. The main ethical objections to genome editing were “tampering with nature” (Iredale et al., 2003; Črne-Hladnik et al., 2009; King et al., 2010) and “playing God” (Macer et al., 1995; Bellenguez et al., 2022).

Studies also agree that genetic technologies should be used to cure disease rather than to increase efficiency. For example, numerous studies show that gene therapy is more likely to be accepted as a treatment or for reducing disease risks than when it is used for non-medical purposes (Robillard et al., 2014) such as enhancement (Wang et al., 2017), physical traits (Macer et al., 1995), appearance-related applications (Napolitano and Ogunseitan, 1999), or individual characteristics (Hendriks et al., 2018). Furthermore, our study was also able to confirm Küchenhoff’s et al. (2022) findings that gene therapy is more likely to be judged morally good when used for serious diseases than for protection against influenza, which was confirmed for both adult and embryonic use. This is also consistent with the literature (van Lieshout and Dawson, 2016).

Interestingly, neither Küchenhoff et al. (2022) nor our study were able to confirm the hypotheses that individuals who have experience with genetic testing evaluate it as morally superior compared to those without experience, or that self-assessed knowledge about genetics influences the moral evaluation of genetic engineering. However, it should be noted that the difference between individuals with experience and those without experience was only narrowly non-significant. Other studies have shown that both education and knowledge influence attitudes toward genetic technologies (Črne-Hladnik et al., 2009; Cebesoy and Öztekin, 2016), while Ganne et al. (2015) also reported no difference in the acceptance of genetic technology in relation to knowledge. One reason why our study could not show a correlation between self-assessed knowledge and acceptance may be related to the fact that the educational level of our study participants was above average. Nevertheless, our research confirmed Küchenhoff’s findings that objective knowledge about genetics is negatively correlated with the rejection of genetic technologies.

The evaluation of genetic testing as being morally better than genome editing could be confirmed as well. However, future studies should look more closely at whether participants understand that a genetic test is not equivalent to a diagnosis, as misconceptions still exist (Maftei and Dănilă, 2022). It is also important to consider that there are many types of genetic tests, including biochemical, cytogenetic, and molecular, and different applications such as predictive and carrier tests (Beery, 2014). Although there is an increasing number of genetic tests available, some of which can be easily purchased online as so-called direct-to-consumer tests (DTC) (Phillips, 2016), there is also a lack of specific European or uniform national regulations (Kalokairinou et al., 2018).

### Limitations

While we made efforts to ensure a diverse sample by including individuals from various social groups and encouraging their social circles to participate in the study, upon comparing our sociodemographic data to the general population in Germany, it appears that our sample is likely to have a higher level of education (Statistisches Bundesamt, 2020). This bias is a known issue, possibly stemming from the lower participation of less educated individuals in scientific projects. Additionally, the recruitment channels we utilized primarily targeted individuals with a specific interest in research projects.

It is important to consider the limitations associated with the online survey format. Online surveys tend to attract respondents who are technologically proficient or have a lot of free time (Wright, 2005; Hunter, 2012), leading to a potential selection bias and skewed results. Moreover, the absence of personal interaction, as seen in face-to-face interviews, restricts the opportunity to explore more detailed or nuanced responses (Ball, 2019). Lastly, technical difficulties, such as slow loading times or issues with the survey software, can frustrate participants and potentially impact response rates.

Therefore, for future projects, we recommend using larger sample sizes and implementing an expanded recruitment program. Convenience samples, in general, tend to be statistically biased due to their composition being predominantly WEIRD (Western, Educated, Industrialized, Rich, and Democratic) (Henrich et al., 2010). Thus, the ability to generalize and make cross-cultural comparisons is limited.

One inherent issue in this study is the potential for respondent fatigue resulting from completing many consecutive scales. The order of these scales was not randomized because our survey instrument does not support this method.

## Conclusion

All five versions of the GTQ can be used as valid research instruments. Follow-up studies should focus on the further improvement of the instrument, e.g., the deletion of item 14, or the deletion of items 28 or 30, as they are highly correlated, making them statistically almost identical. Instead, an item from the area of privacy, data security, or justice distribution should be added to improve the factor structure. Further studies should also investigate the reasons behind each moral judgment, as this is the only way to determine whether the evaluation is based on a lack of knowledge and misconceptions such as a belief in genetic determinism or a religious standpoint. By having a variety of instruments, we can use the most appropriate scale for questions ranging from genetics to applied ethics. Moreover, it would be desirable to translate the instrument into other languages to explore cultural differences in the evaluation of genetic technologies.

## Data Availability

The datasets presented in this study can be found in online repositories. The names of the repository/repositories and accession number(s) can be found below: OSF (Genetic Technologies Questionnaire – German version) https://osf.io/4gmf9/.

## Abbreviations

CTQ, Conventional Technologies Questionnaire; DNA, deoxyribonucleic acid; GTQ, Genetic Technologies Questionnaire; GTQ-H, GTQ-Human; GTQ-MS, GTQ-Moral Status; KMGG, Knowledge about Modern Genetics and Genomics; KMO, Kaiser-Meyer-Olkin coefficient, PCA, Principal Component Analysis; STROBE, Strengthening the Reporting of Observational Studies in Epidemiology; WEIRD, Western, Educated, Industrialized, Rich, and Democratic; WMW, Wilcoxon-Mann-Whitney test.
