# Supplementary material for: Moral judgment of genetic technologies: validation of the genetic technologies questionnaire in the German-speaking population
Source: Front Genet. 2025 Aug 1;16:1620962. doi: 10.3389/fgene.2025.1620962 (PMC12353722; doi:10.3389/fgene.2025.1620962)
Supplement: Supplementary file 2 [file Supplementaryfile3.docx]

Supplementary Material

Table 1. Factor loadings of the Principal Component Analysis of the GTQ-H

| Nr. | Item | **F_I_** - gene editing | **F_II_** - genetic testing | **F_III_** - data privacy |
| --- | --- | --- | --- | --- |
| 18 | Genome editing of human adults to protect them against influenza is | 0.78 |  |  |
| 19 | Changing the genome of human embryos to ensure they will not get influenza is … | 0.78 |  |  |
| 22 | Using genome editing to enhance the cognitive development of human embryos in underprivileged families is … | 0.72 |  |  |
| 15 | Using genome editing on consenting adults to enhance their cognitive performance is … | 0.71 |  |  |
| 16 | Changing the genomes of human embryos for medical research without destroying them is… | 0.67 |  |  |
| 17 | Changing the genomes of human embryos to ensure they will not develop a fatal disease is … | 0.64 |  |  |
| 2 | Prescribing genetic tests for healthy women in order to identify markers for breast cancer is … |  | 0.76 |  |
| 4 | Performing genetic tests on consenting adult humans for medical research is … |  | 0.72 |  |
| 3 | Using genetic tests to determine if one carries markers for hereditary diseases before deciding to conceive a child is … |  | 0.71 |  |
| 12 | Using public health funds on expensive gene therapies is … |  | 0.66 |  |
| 21 | Testing for the risk of genome editing on consenting adults is … |  | 0.63 |  |
| 1 | Genetic testing to determine the risk of Down’s syndrome for an embryo in utero is… |  | 0.50 |  |
| 20 | Using risky genome editing therapies for the medical treatment of cancer patients is … |  | 0.49 |  |
| 11 | For insurers, requesting genetic tests from healthy adults in order to assess their health risks is … |  |  | 0.72 |
| 9 | Consider a patient with a hereditary disease who has a sibling with similar genes. For the doctor, informing the sibling of the patient’s disease despite privacy concerns is… |  |  | 0.69 |
| 10 | Supporting genetic testing despite privacy concerns is… |  |  | 0.63 |
| 13 | Taking into account the genetic profile of applicants with respect to genetic diseases when hiring a kindergarten teacher is… |  |  | 0.62 |
|  | Explained variance of the rotated factors. | 21.8% | 19.8% | 12.5% |
