# Supplementary material for: Moral judgment of genetic technologies: validation of the genetic technologies questionnaire in the German-speaking population
Source: Front Genet. 2025 Aug 1;16:1620962. doi: 10.3389/fgene.2025.1620962 (PMC12353722; doi:10.3389/fgene.2025.1620962)
Supplement: Supplementary file 3 [file Supplementaryfile2.docx]

**„Genetic Technologies Questionnaire“ (Küchenhoff et al. 2022)**

|  | **Englisch (Küchenhoff et al. 2022)** | **German** |
| --- | --- | --- |
| 1 | Genetic testing to determine the risk of Down’s syndrome for an embryo in utero is… | Genetische Tests zur Bestimmung des Risikos von Down-Syndrom (Trisomie 21) bei einem Embryo in der Gebärmutter sind ... |
| 2 | Prescribing genetic tests for healthy women in order to identify markers for breast cancer is … | Das Verschreiben genetischer Tests zur Feststellung von Brustkrebsmarkern bei gesunden Frauen ist ... |
| 3 | Using genetic tests to determine if one carries markers for hereditary diseases before deciding to conceive a child is … | Das Durchführen von genetischen Tests für die Bestimmung von Erbkrankheiten, bevor man sich entscheidet, ein Kind zu zeugen, ist ... |
| 4 | Performing genetic tests on consenting adult humans for medical research is … | Das Durchführen von genetischen Tests zwecks medizinischer Forschung bei Erwachsenen, die ihr Einverständnis dazu gegeben haben, ist ... |
| 5 | Conducting harmless genetic tests on animals for scientific research is… | Das Durchführen harmloser genetischer Tests an Tieren für die wissenschaftliche Forschung ist ... |
| 6 | *Optimizing the breeding of farm animals through genetic testing is…* | *Die Zucht von Nutztieren durch genetisches Testen zu optimieren, ist ...* |
| 7 | *Performing invasive genetic tests on wild plants to monitor and conserve ecosystems is…* | *Die Durchführung invasiver genetischer Tests an Wildpflanzen zur Überwachung und Erhaltung des Ökosystems ist ...* |
| 8 | *Genetic testing of crops to improve them for farming is…* | *Genetische Tests an Nutzpflanzen, um sie für die Landwirtschaft zu verbessern, sind ...* |
| 9 | Consider a patient with a hereditary disease who has a sibling with similar genes. For the doctor, informing the sibling of the patient’s disease despite privacy concerns is… | Stellen Sie sich eine/-n Patient:in mit einer Erbkrankheit vor, die/der ein Geschwisterteil mit ähnlichen Genen hat. Das Informieren des Geschwisterteils über die Krankheit des Patienten seitens des Arztes trotz Schweigepflicht ist ... |
| 10 | Supporting genetic testing despite privacy concerns is… | Genetisches Testen trotz Datenschutzbedenken ist ... |
| 11 | For insurers, requesting genetic tests from healthy adults in order to assess their health risks is … | Das Anfordern genetischer Tests gesunder Erwachsener seitens der Versicherung zur Einschätzung gesundheitlicher Risiken ist ... |
| 12 | *Using public health funds on expensive gene therapies is …* | *Der Einsatz öffentlicher Gesundheitsgelder für teure Gentherapien ist ...* |
| 13 | Taking into account the genetic profile of applicants with respect to genetic diseases when hiring a kindergarten teacher is… | Die Berücksichtigung des genetischen Profils von Bewerber:innen im Hinblick auf Erbkrankheiten bei der Einstellung von Erzieher:innen im Kindergarten ist ... |
| 14 | Mitigating a criminal sentence due to the offender’s genetic predisposition is … | Die Strafmilderung aufgrund der genetischen Veranlagung des/der Straftäter:in ist ... |
| 15 | *Using genome editing on consenting adults to enhance their cognitive performance is …* | *Die Veränderung des Erbguts (Genome Editing) zur Verbesserung der kognitiven Leistung bei Erwachsenen, die ihr Einverständnis dazu gegeben haben, ist ...* |
| 16 | *Changing the genomes of human embryos for medical research without destroying them is…* | *Die Veränderung des Erbguts bei menschlichen Embryonen für die medizinische Forschung, ohne sie anschließend zu vernichten, ist ...* |
| 17 | *Changing the genomes of human embryos to ensure they will not develop a fatal disease is …* | *Die Veränderung des Erbguts bei menschlichen Embryonen zur Vermeidung von schwerwiegenden oder tödlichen Krankheiten ist ...* |
| 18 | ***Genome editing of human adults to protect them against influenza is …*** | ***Die Veränderung des Erbguts von Erwachsenen zum Schutz vor Influenza ist*** |
| 19 | ***Changing the genome of human embryos to ensure they will not get influenza is …*** | ***Die Veränderung des Erbguts bei menschlichen Embryonen zum Schutz vor Influenza ist ...*** |
| 20 | *Using risky genome editing therapies for the medical treatment of cancer patients is …* | *Der Einsatz riskanter erbgutverändernder Therapien zur medizinischen Behandlung von Krebspatient*innen ist ...* |
| 21 | *Testing for the risk of genome editing on consenting adults is …* | *Das Testen des Risikos einer Erbgutveränderung bei Erwachsenen mit deren Einverständnis ist ...* |
| 22 | *Using genome editing to enhance the cognitive development of human embryos in underprivileged families is …* | *Die Veränderung des Erbguts zur Verbesserung der kognitiven Entwicklung menschlicher Embryonen in benachteiligten Familien ist ...* |
| 23 | ***Changing the genome of farm animals in order to improve their wellbeing is …*** | ***Das Erbgut von Nutztieren zu verändern, um deren Wohlbefinden zu verbessern, ist ...*** |
| 24 | ***Editing the genome of farm animals to reduce costs without harming them is …*** | ***Das Erbgut von Nutztieren zur Kosteneinsparung zu verändern, ohne ihnen zu schaden, ist ...*** |
| 25 | *Editing the genome of crops in order to fight world poverty is …* | *Die Erbgutveränderung von Saatgut zur Bekämpfung der globalen Armut ist ...* |
| 26 | *Editing the genome of foods to improve their taste is …* | *Die Erbgutveränderung von Lebensmitteln zur Verbesserung ihres Geschmacks ist ...* |
| 27 | *Editing the genome of animals to make it possible for animal organs to be transplanted to humans is …* | *Die Erbgutveränderung bei Tieren, um deren Organe für Transplantationen bei Menschen nutzen zu können, ist ...* |
| 28 | Editing the genome of crops to improve their nutritional value is… | Die Erbgutveränderung von Saatgut zur Verbesserung des Nährwertes ist ... |
| 29 | *Editing the genome of wild animals to make them immune against certain diseases is …* | *Die Erbgutveränderung von Wildtieren, um sie gegen bestimmte Krankheiten immun zu machen, ist ...* |
| 30 | ***Editing the genome of plants to improve crops for farming is…*** | ***Die Erbgutveränderung von Pflanzen zur Verbesserung des Saatgutes ist ...*** |

Italicized items are included in the GTQ20, bold items are included in the GTQ5.

**Conventional Technologies Questionnaire 5 (CTQ5)**

|  | **English (Küchenhoff et al. 2022)** | **Greek** |
| --- | --- | --- |
| 1 | Vaccinating human adults to protect them against influenza is … | Die Impfung Erwachsener zum Schutz vor Influenza ist ... |
| 2 | Using vaccination on human embryos to ensure they will not get influenza is … | Der Einsatz von Impfungen bei menschlichen Embryonen zum Schutz vor Influenza ist ... |
| 3 | Changing the hormones of farm animals in order to improve their wellbeing is … | Hormonveränderungen von Nutztieren, um ihr Wohlbefinden zu verbessern, sind ... |
| 4 | Changing the hormone balance of farm animals to reduce costs without harming them is … | Veränderung des Hormonhaushalts von Nutztieren, um Kosten zu senken, ohne ihnen zu schaden, ist ... |
| 5 | Selectively breeding plants to improve crops for farming is… | Selektive Pflanzenzüchtung zur Verbesserung des Saatguts für die Landwirtschaft ist ... |
